# Extracellular *N*-Acetylaspartate in Human Traumatic Brain Injury

**DOI:** 10.1089/neu.2015.3950

**Published:** 2016-02-15

**Authors:** Richard J. Shannon, Susan van der Heide, Eleanor L. Carter, Ibrahim Jalloh, David K. Menon, Peter J. Hutchinson, Keri L.H. Carpenter

**Affiliations:** ^1^ Division of Neurosurgery, Department of Clinical Neurosciences, University of Cambridge, Cambridge, United Kingdom.; ^2^ Wolfson Brain Imaging Centre, Department of Clinical Neurosciences, University of Cambridge, Cambridge, United Kingdom.; ^3^ Division of Anaesthesia, Department of Medicine, University of Cambridge, Cambridge, United Kingdom.

**Keywords:** brain metabolism, microdialysis, *N*-acetylaspartate, traumatic brain injury (human)

## Abstract

*N*-acetylaspartate (NAA) is an amino acid derivative primarily located in the neurons of the adult brain. The function of NAA is incompletely understood. Decrease in brain tissue NAA is presently considered symptomatic and a potential biomarker of acute and chronic neuropathological conditions. The aim of this study was to use microdialysis to investigate the behavior of extracellular NAA (eNAA) levels after traumatic brain injury (TBI). Sampling for this study was performed using cerebral microdialysis catheters (M Dialysis 71) perfused at 0.3 μL/min. Extracellular NAA was measured in microdialysates by high-performance liquid chromatography in 30 patients with severe TBI and for comparison, in radiographically “normal” areas of brain in six non-TBI neurosurgical patients. We established a detailed temporal eNAA profile in eight of the severe TBI patients. Microdialysate concentrations of glucose, lactate, pyruvate, glutamate, and glycerol were measured on an ISCUS clinical microdialysis analyzer. Here, we show that the temporal profile of microdialysate eNAA was characterized by highest levels in the earliest time-points post-injury, followed by a steady decline; beyond 70 h post-injury, average levels were 40% lower than those measured in non-TBI patients. There was a significant inverse correlation between concentrations of eNAA and pyruvate; eNAA showed significant positive correlations with glycerol and the lactate/pyruvate (L/P) ratio measured in microdialysates. The results of this on-going study suggest that changes in eNAA after TBI relate to the release of intracellular components, possibly due to neuronal death or injury, as well as to adverse brain energy metabolism.

## Introduction

*N*-acetylaspartate (NAA) is one of the most abundant amino acid derivatives present in the central nervous system (CNS), where it is thought to play a significant functional role.^1^ NAA was first reported in 1956, though its function, complex synthesis, and metabolism remain poorly understood.^2^ NAA is synthesized in the neuronal cytosol from acetic acid and L-aspartic acid by the endoplasmic reticulum-associated enzyme *N*-acetyltransferase 8-like.^3–6^ Upon release, NAA is taken up by oligodendrocytes for hydrolysis to acetic acid and L-aspartic acid via the enzyme aspartoacylase (ASPA).^7^ Input by astrocytes is required for ASPA to catalyze this reaction.^8^ NAA biosynthesis and processing is thus linked to the availability of acetyl coenzyme A (acetyl-CoA), cell adenosine triphosphate (ATP) stores, overall energy state, and mitochondrial phosphorylating capacity.^9^

The role of NAA in myelin lipid synthesis, particularly in early development, is well established. The acetic acid from NAA becomes incorporated into CNS myelin.^10^ Inherited *ASPA* gene mutations result in Canavan disease, showing ASPA deficiency in oligodendrocytes, abnormally high brain NAA levels, deficient axonal myelin sheath development, and spongy degeneration of white matter.^11–14^ NAA plays a vital role as a precursor for the neurotransmitter *N*‐acetylaspartylglutamate.^15^ In addition, NAA traps excess aspartate and thus transports amino group nitrogen from mitochondria to the cytoplasm.^16,17^ NAA may provide a store of energy metabolism precursors in neurons, astrocytes and oligodendrocytes in the absence of a sufficient store of glycogen,^3^ and may be involved in maintaining neuronal osmolytic balance.^1,18,19^

In proton magnetic resonance spectroscopy (^1^H-MRS) of brain, NAA shows as a large, prominent peak. Relative to healthy controls, NAA is significantly reduced in neurological conditions, such as stroke, Alzheimer's disease, dementia, Huntington's disease, multiple sclerosis, and epilepsy.^20,21^ Although mechanisms remain unclear, diminished NAA is considered a non-invasive ^1^H-MRS marker for neuronal injury, morbidity, or metabolic dysfunction.^22,23^ Severe traumatic brain injury (TBI) results in a reduction in the brain NAA levels (measured ex vivo by high-performance liquid chromatography [HPLC]) in animal models, even in areas remote from the focal injury.^6^ This reduction recovered to basal level in diffuse neuronal injury models at a rate inversely proportional to injury severity and in correlation with brain energy state.^6^ Partial recovery of brain NAA levels was reported using ^1^H-MRS in a small follow-up study of acute brain damage patients.^24^

Microdialysis (MD) is a well-established technique enabling both clinical monitoring at the bedside and neurochemical analysis in the laboratory, for extracellular molecules from the human brain.^25,26^ Extracellular NAA (eNAA) levels in rats with diffuse TBI did not differ significantly from sham baseline in brain microdialysates and whole brain tissue, and in brain ^1^H-MRS.^27,28^ Recently, TBI patients with microdialysis were studied by Belli and colleagues., who reported that a lower average eNAA concentration in the 9 d following injury was associated with poorer clinical outcome, whereas a peak in eNAA at around 5 d post-injury was associated with patient survival.^23^ In the present study, we have measured eNAA in 30 TBI patients with microdialysis. We have analyzed the detailed temporal profile of eNAA concentrations in eight of these TBI patients, along with other biochemical markers. We have compared eNAA levels in TBI patients who received glucose-supplemented microdialysis fluid and those who did not to establish the cellular response to a localized improvement in fuel supply. We also have compared TBI eNAA levels with those from “normal” brain in six non-TBI neurosurgical patients.

## Methods

### Subjects

The study was approved by the Cambridge Local Research Ethics Committee and assent obtained from the next of kin. All TBI subjects were patients in the Neurosciences Critical Care Unit (NCCU) in Addenbrooke's Hospital, Cambridge. Patients were older than 16 years of age with TBI requiring ventilation and intracranial pressure (ICP) monitoring. The major exclusion criterion was deranged clotting and/or low platelets, precluding the placement of a microdialysis catheter. None of the TBI patients had any significant previous neurological conditions or a family history of neuro-degenerative disease.

Patients undergoing a craniotomy for resection of benign brain tumors, enabling microdialysis catheters to be safely placed into radiographically normal brain, were selected as control subjects. The microdialysis catheters were placed via the craniotomy and tunneled under the scalp of the patient. Control patients were awake for the duration of the microdialysis perfusion.

### Microdialysis

A total of 30 TBI patients were monitored using microdialysis, which was started as soon as possible following admission to the NCCU. Microdialysis catheters (CMA71, 100 kDa molecular weight cut-off respectively, 10-mm membrane; M Dialysis AB, Stockholm, Sweden) were inserted into the cerebral frontal parenchyma of patients, together with an ICP transducer (Codman, Raynham, MA) using a triple-lumen cranial access device (Technicam, Newton Abbot, UK). One of the 30 patients studied received two catheters at different cranial sites. The catheters were perfused with either regular CNS perfusion fluid (NaCl, 147 mM; KCl, 2.7 mM; CaCl_2_, 1.2 mM; and MgCl_2_, 0.85 mM in water; M Dialysis AB), or CNS perfusion fluid supplemented with 4 mM 1,2-^13^C_2_ glucose (Pharmacy Manufacturing Unit, the Ipswich Hospital NHS Trust, Ipswich, UK). Patients with two catheters were treated with two types of perfusion fluid: one with and one without glucose supplementation. A perfusion flow rate of 0.3 μL min^−1^ was provided using a CMA106 pump (M Dialysis AB). Collection vials were changed hourly and analyzed at the bedside for glucose, lactate, pyruvate and glutamate or glycerol, using a CMA600 or ISCUSFlex microdialysis analyzer (M Dialysis AB) using automated enzymatic colorimetric assays as part of routine multimodality monitoring. Microdialysates were then stored at −80°C for subsequent analysis by HPLC to determine amino acids and NAA. Microdialysates from each patient were pooled into either 8 h periods (normal CNS perfusion fluid) or 24 h periods (CNS perfusion fluid supplemented with glucose) for HPLC analysis.

The six benign tumor resection patients were monitored using microdialysis, which commenced immediately after surgery was completed. The catheters were perfused with CNS perfusion fluid (as above) supplemented with glucose (as above) at a flow rate of 0.3 μL min^−1^. Collection vials were changed hourly and analyzed with the ISCUS at 4-h intervals. Microdialysates were then stored at −80°C for subsequent analysis by HPLC to determine amino acids and NAA. Microdialysates from each patient were pooled into 24 h periods for HPLC analysis.

### Determination of N-acetylaspartate (NAA) by HPLC

Microdialysate samples were diluted using the ratio of 5 μL of microdialysate with 10 μL of water prior to HPLC analysis on an Agilent 1100 series HPLC (Agilent Technologies, Waldbronn, Germany) using an adapted version of methodology published previously.^29^ The HPLC system comprised of a binary pump, refrigerated auto-sampler (at 10°C), and ultraviolet detector (at 210 nm), with a ChemStation data system (Agilent). The column (maintained at 20°C) was a Phenomenex Hyperclone C18 (150 mm × 2 mm, particle size 3 μm, pore size 100 Å; Phenomenex, Torrance, CA), with a Phenomenex SecurityGuard Luna C18(2) guard cartridge. The mobile phase (0.25 mL min^−1^) was continuously vacuum-degassed. Using the auto-sampler, each pre-diluted microdialysate sample (2 μL) was injected onto the HPLC column. Separation was achieved using isocratic elution. Mobile phase consisted of 25 mM potassium phosphate in water, 2.8 mM tetrabutylammonium hydroxide (Sigma-Aldrich, Gillingham, Dorset, UK), and 1.25% v/v methanol, pH 7.0. The run time was 15 min, and after every four samples, the column was washed with a mobile phase consisting of 100 mM potassium phosphate in water, 2.8 mM tetrabutylammonium hydroxide, and 30% v/v methanol, pH 5.5. Before injection of the next four samples, the column was equilibrated with the original mobile phase for 1 h. Quantitation was by peak areas relative to external standards of NAA (Sigma-Aldrich). NAA typically eluted at 6.6 mins. The limit of quantification of the HPLC method is 1.8 μM.

### Statistical analysis

Statistical analyses were performed using SPSS21 software (IBM SPSS Statistics, Armonk, NY) included non-parametric tests (Mann-Whitney *U*-test). Relationships between eNAA and time after injury or operation, and eNAA and other biomarkers were explored using linear regression, with Spearman's rank correlation coefficient *r* and analysis of variance (ANOVA) *p* values. A pre-selected *p* value of 0.05 was considered statistically significant.

## Results

### Demography

A total of 30 severe TBI patients and six non-TBI patients were studied. Extracellular NAA concentrations were monitored in a detailed temporal profile in eight of the TBI patients (five male and three female), ages 20 to 69 years (median, 56 years). Extracellular NAA was also measured in 22 additional severe TBI patients (17 male and five female), ages 16 to 64 years (median, 28 years). The latter group of patients received either non-supplemented perfusion fluid, or 4 mM 1,2-^13^C_2_ glucose-supplemented perfusion fluid via the microdialysis catheter, while simultaneously collecting the emerging microdialysates for analysis. One of the 22 patients received two microdialysis catheters at different locations, through which the concentration of glucose also varied. Radiographically normal–appearing brain was studied as a means of comparison using the aforementioned ^13^C-labeled microdialysis method in six patients (ages 42–73 years; three male, three female) undergoing surgery for benign brain tumors. The 22 additional TBI patients, and the six normal brain control patients were the same patients reported in a previous study within our research group.^30^ Patient demography and the types of perfusion fluid for each patient are presented in Table 1.

**Table 1.** Patient Demography and Types of Perfusion Fluid

*TBI Time-course Patient*	*Age*	*Sex*	*Injury mechanism*	*GCS at scene/15*	*Microdialysis start time after injury / h*	*GOS score*	*Perfusion fluid - 1,2 -*^13^*C_2_ glucose concentration*
1	27	M	Fall	8	48	3	0
2	28	M	RTC	3	24	2	0
3	29	M	RTC	4	72	3	0
4	30	F	RTC	3	48	3	0
5	31	F	RTC	14	24	3	0
6	32	F	RTC	12	35	3	0
7	33	M	Assault	9	20	5	0
8	34	M	Fall	11	18	3	0

*TBI patient*	*Age*	*Sex*	*Injury mechanism*	*GCS at scene/15*	*Microdialysis start time after injury / h*	*GOS score*	*Perfusion fluid - 1,2 -*^13^*C_2_ glucose concentration*	*Number of sampling periods*
9	26	F	RTC	6	17	4	4 mM	2
10	27	M	RTC	7	45	5	4 mM	2
11	53	F	RTC	6	41	3	4 mM	2
12	59	F	RTC	4	60	3	4 mM	2
13	16	F	RTC	4	47	5	4 mM	2
14	37	M	RTC	8	49	5	4 mM	1
15	28	M	RTC	3	104	5	4 mM	2
16	20	M	RTC	4	132	4	4 mM	1
17	20	M	RTC	5	51	NA	4 mM	1
18	25	M	RTC	8	26	4	4 mM	1
19	20	M	RTC	4	88	5	4 mM	1
20 (A)	27	M	Assault	5	17	3	4 mM	1
20 (B)							Unsupplemented	1
21	61	M	RTC	11	27	4	Unsupplemented	1
22	49	M	Assault	NA	41	4	Unsupplemented	1
23	47	M	Fall	7	65	4	4 mM	4
24	27	M	Fall	NA	17	3	Unsupplemented	1
25	27	M	RTC	NA	27	N/A	4 mM	3
26	28	F	Fall	11	104	3	4 mM	1
27	29	M	RTC	NA	16	3	4 mM	1
28	52	M	Fall	8	15	5	4 mM	1
29	64	M	Fall	NA	98	1	4 mM	1
30	49	M	Fall	3	20	2	4 mM	1

*Normal brain subject*	*Age*	*Sex*	*Benign tumor type*	*Perfusion fluid - 1,2 -*^13^*C_2_ glucose concentration*	*Number of sampling periods*
1	60	F	Meningioma	4 mM	1
2	59	M	Colloid cyst	4 mM	2
3	59	M	Meningioma	4 mM	1
4	73	M	Meningioma	4 mM	2
5	50	F	Meningioma	4 mM	1
6	42	F	Meningioma	4 mM	1

A total of 30 TBI patients and non-TBI neurosurgical patients were recruited. Twenty-two TBI patients underwent a period of microdialysis with either unsupplemented perfusion fluid, or perfusion fluid containing 4 mM 1,2-^13^C_2_ glucose. Another eight patients who received unsupplemented perfusion fluid were monitored for eNAA over a period of ≤240 h. Six non-TBI patients undergoing craniotomies for benign tumors underwent microdialysis with the catheter placed in radiographically normal brain, all of whom received perfusion fluid supplemented with 4 mM 1,2-^13^C_2_ glucose.

TBI, traumatic brain injury; GCS, Glasgow Coma Scale score; GOS, Glasgow Outcome Scale; M, male; RTC, road traffic collision; F, female.

### Temporal profile of eNAA concentration

**FIG. 1.**
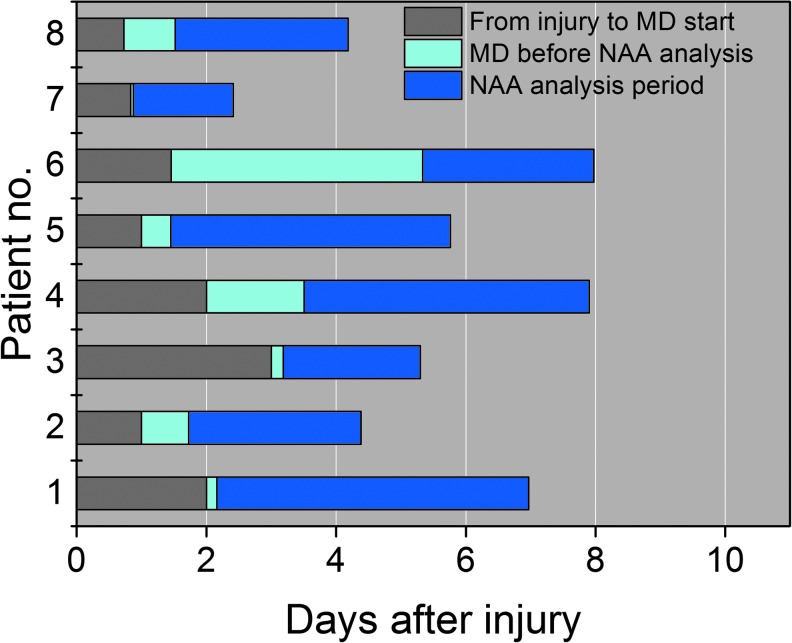
Time lines for sampling and analysis of microdialysis samples from eight traumatic brain injury patients during the time-course study. MD, microdialysis; NAA, *N*-acetylaspartate. Color image is available online at www.liebertpub.com/neu

**FIG. 2.**
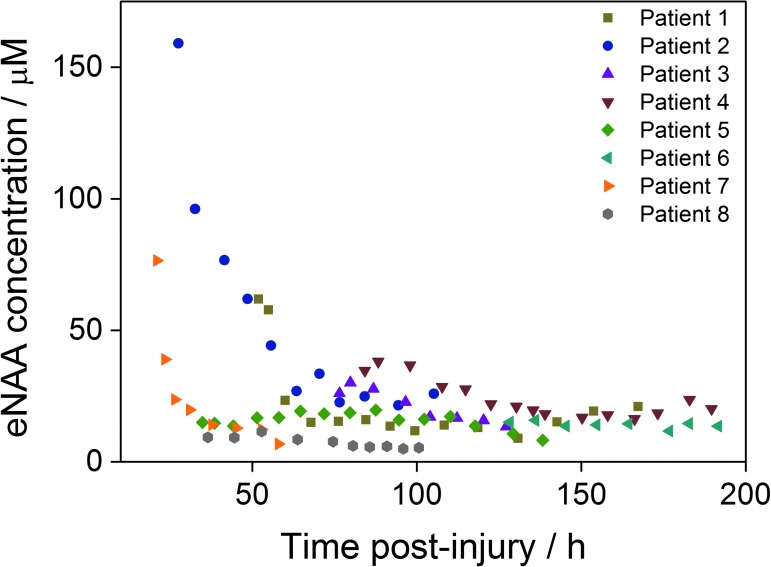
The temporal profile of extracellular *N*-acetylaspartate (eNAA) concentration in microdialysis samples from eight traumatic brain injury patients in relation to time since injury. Each data-point represents one 8 h pooled sample. Results are for *n* = 61 data-points, Spearman *r* = −0.28, *p* = 0.032. Color image is available online at www.liebertpub.com/neu

The time-course of eNAA concentration was determined in eight patients for an average monitoring period ranging from 36 h to 75 h after injury, the exact times of which are shown in Figure 1. The average time monitored in each patient was 39 h, and the average time for every data-point was 8 h. Figure 2 shows the average eNAA concentration for each data-point for the eight patients monitored, plotted versus post-injury time. A steep decline in the concentration of eNAA occurred early after injury, particularly in Patients 1, 2, and 7, where monitoring began <50 h rather than later after injury (Fig. 2). Taking together the data from all eight patients, there was a significant inverse correlation between eNAA concentration and time post-injury (Fig. 2). A significant difference also was observed between eNAA concentrations before and after 96 h post-injury (Mann-Whitney, *p* = 0.017). None of the patients who took part in this study died as a result of their injuries; however, it was noted that the highest concentration of eNAA was obtained in the first sample taken after injury (Fig. 2) from the patient with the worst clinical outcome (Glasgow Outcome Scale [GOS] score 2).

### Effect of glucose supplementation through microdialysis catheter

**FIG. 3.**
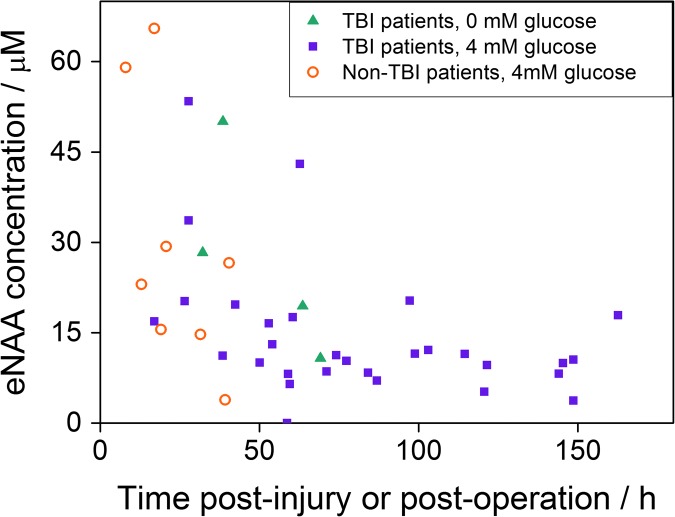
The concentrations of extracellular *N*-acetylaspartate (eNAA) measured in microdialysates from 22 traumatic brain injury (TBI) patients and six non-TBI patients plotted vs. time post-injury (TBI patients) or time post-operation (non-TBI patients). Of the 22 TBI patients, four received non-supplemented perfusion fluid, and 15 received perfusion fluid supplemented with 4 mM glucose. The patient groups are differentiated by symbols as indicated: filled triangles, TBI patients without glucose; filled squares, TBI patients with 4 mM glucose; open circles, non-TBI patients with 4 mM glucose. Each data-point represents one 8 h pooled sample. There was a significant inverse relationship for NAA vs. post-injury time for the group of 15 TBI patients receiving perfusion fluid supplemented with 4 mM glucose (*n* = 30 data-points, Spearman *r* = −0.396, *p* = 0.030). Some of the 15 TBI patients with 4 mM glucose had more than one pooled sample available, making 30 data-points for this group (as shown in Table 1). Color image is available online at www.liebertpub.com/neu

The effect of localized fuel (glucose) supplementation via the microdialysis catheter on the eNAA concentration was determined in 22 TBI patients. Of these, four were treated with non-supplemented perfusion fluid, and the remaining 17 were treated with perfusion fluid containing 4 mM glucose. The concentrations of eNAA plotted versus time post-injury are shown in Figure 3, where the data-points are differentiated according to the composition of the perfusion fluid. A significant inverse relationship between time post-injury and eNAA concentration was observed for patients receiving microdialysis with perfusion fluid supplemented with 4 mM glucose (Fig. 3).

### Microdialysis of non-TBI patients with glucose supplementation

**FIG. 4.**
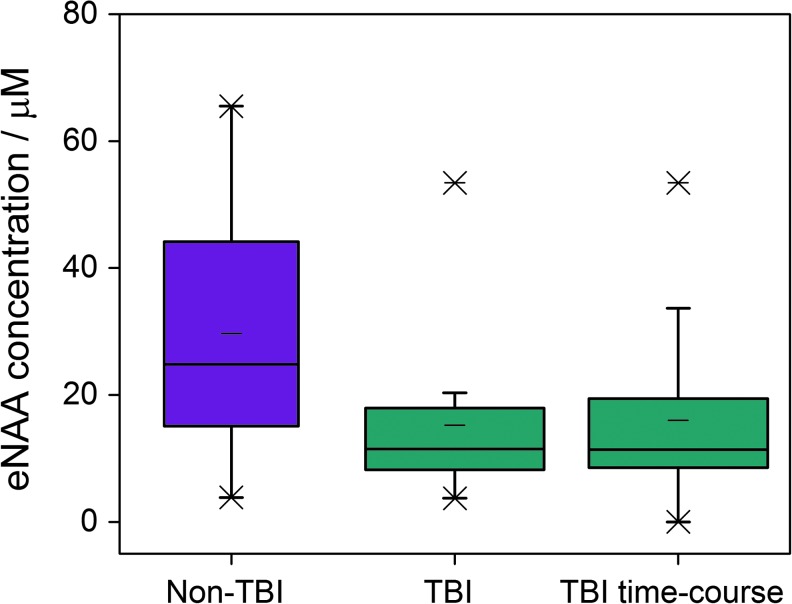
The extracellular *N*-acetylaspartate (eNAA) concentrations in microdialysis samples from six non- traumatic brain injury (TBI) patients vs. those measured in 30 non-TBI patients at time-points taken after 96 h post-injury. The 30 TBI patients are divided into two groups; the TBI patients of which four received non-supplemented perfusion fluid and 15 received perfusion fluid supplemented with 4 mM glucose, and the time-course TBI patients who received non-supplemented perfusion fluid. Graphs were plotted from 24 h pooled samples for the non-TBI and TBI patients (*n* = 6 and 22, respectively), and 8 h pooled samples from the TBI time-course patients (*n* = 8). Color image is available online at www.liebertpub.com/neu

Extracellular NAA concentrations were measured in radiographically normal areas of brain in six patients who underwent surgery for benign brain tumors as non-TBI controls for comparison with eNAA data from TBI patients. All six non-TBI patients received perfusion fluid supplemented with 4 mM glucose through the MD catheter. The eNAA concentrations obtained from the non-TBI patients in relation to time since operation are shown alongside the eNAA levels measured in TBI patients in Figure 3. No significant correlation was found between time post-operation and eNAA concentration for non-TBI patients (*n* = 9, Spearman *r* = −0.583, *p* = 0.099). The difference between eNAA levels in the TBI patients (median 11.3, interquartile range [IQR] 8.8-19.1 μmol L^−1^) and non-TBI patients (median 24.8, IQR 15.3-36.7 μmol L^−1^) also was not significant (Mann-Whitney, *p* = 0.113). However, a clear difference was observed between eNAA concentrations between non-TBI and TBI patients at time-points 96 h+ post-injury (Fig. 4).

### Relationship between NAA and other microdialysis analytes

**FIG. 5.**
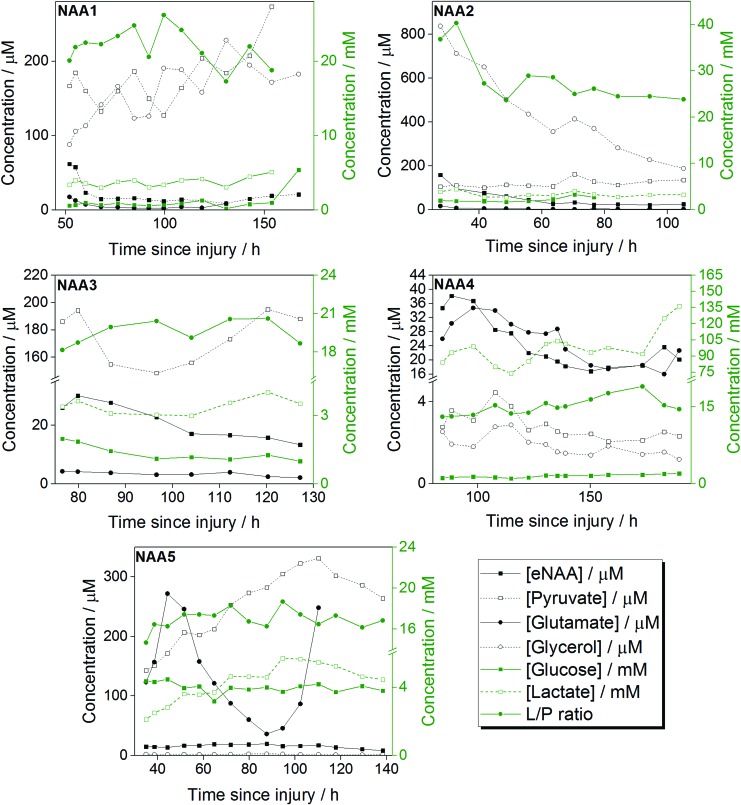
Illustrative examples of the temporal profile of extracellular *N*-acetylaspartate (eNAA; μM), plotted alongside the concentrations of pyruvate, glutamate and glycerol (in μM), glucose and lactate (in mM), and the lactate/pyruvate (L/P) ratio, all measured in brain microdialysates. Extracellular NAA was quantified by high-performance liquid chromatography and the other analytes by ISCUS analysis. These data are from Patients 1–5 of the time-course study. Concentrations are plotted vs. time post-injury. Each data-point represents one 8 h pooled sample. Color image is available online at www.liebertpub.com/neu

**FIG. 6.**
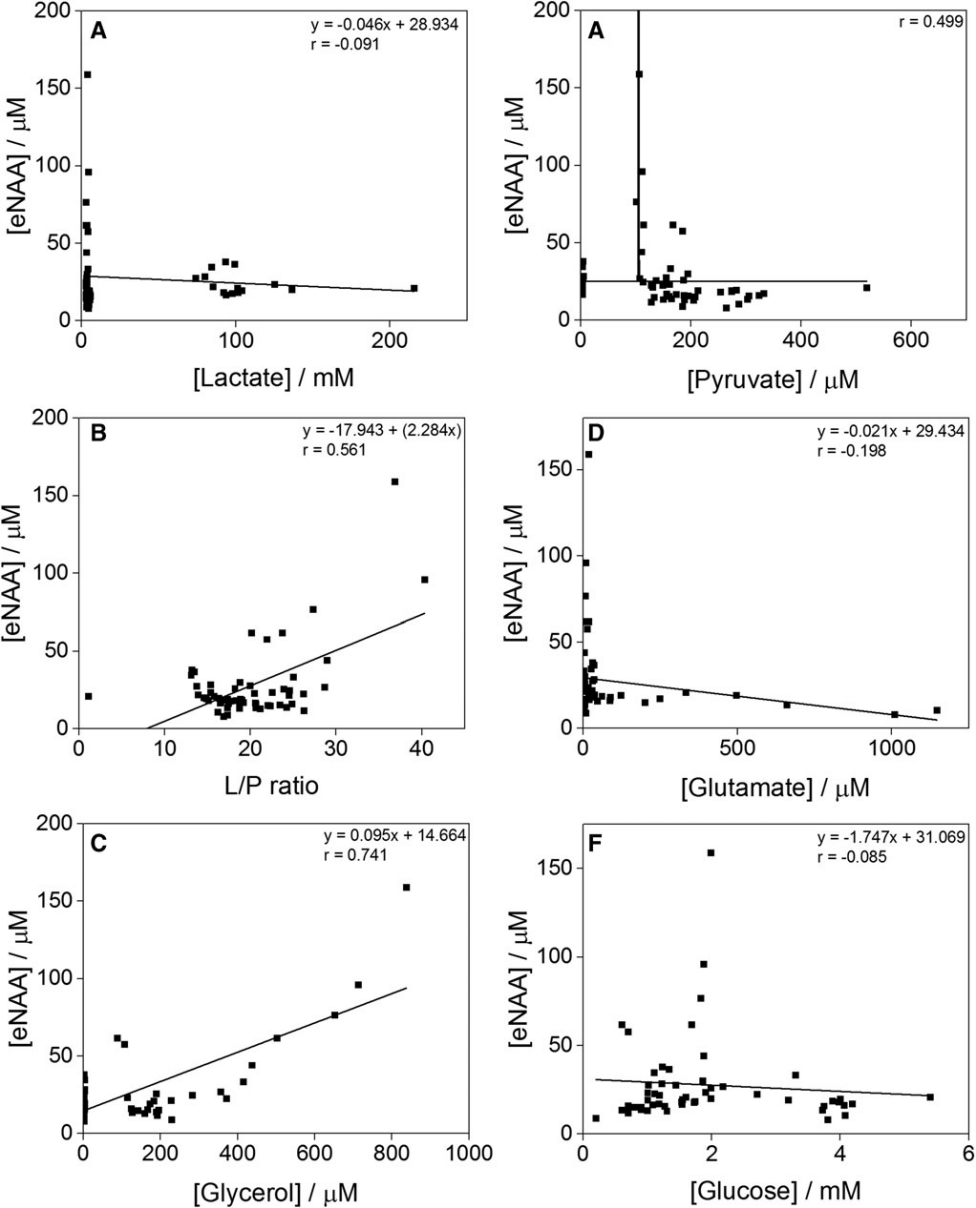
Graphs illustrating the relationships between extracellular *N*-acetylaspartate (eNAA) concentrations and those of lactate **(A)**, pyruvate **(B)**, the lactate/pyruvate (L/P) ratio **(C)**, glutamate **(D)**, glycerol **(E)** and glucose **(F)**, all measured in microdialysates from the eight time-course traumatic brain injury patients (pooled data). Extracellular NAA was quantified by high-performance liquid chromatography and the other analytes by ISCUS analysis. Each data-point represents one 8 h pooled sample. There was a significant inverse correlation between eNAA and pyruvate (*n* = 57, Spearman *r* = −0.418, *p* = 0.001; Fig. 5B), a strong and significant positive linear relationship (*n* = 57, Pearson *r* = 0.561, *p* < 0.00001) between eNAA and the L/P ratio (Fig. 5C), and a significant positive correlation between eNAA and glycerol (*n* = 49, Spearman *r* = 0.360, *p* = 0.011; Fig. 5E).

The concentrations of neurological analytes of clinical relevance (glucose, pyruvate, lactate, glutamate, and glycerol) were determined in the microdialysis fluid of the eight time-course TBI patients alongside their eNAA levels. Figure 5 shows the individual analyte and eNAA profiles of five of these patients throughout the monitoring period. The data-points from all eight patients were pooled for each of the analytes studied, and their relationship with eNAA levels investigated. Bivariate scattergrams for the individual analytes are shown in Figure 6. A significant inverse correlation was established between eNAA and pyruvate (Fig. 6B), while there was a significant positive correlation between eNAA and glycerol (Fig. 6E). There was also a strong and significant positive linear relationship between eNAA and the L/P ratio (Fig. 6C). It was noted that the highest concentration of pyruvate and the highest L/P ratios were observed in Patient 2, in whom both these parameters showed a steady decrease throughout the monitoring period. This same patient was seen as having the highest initial eNAA concentration of all the patients studied, as well as notably the worst clinical outcome (GOS score 2).

## Discussion

This study has shown that elevated eNAA levels after TBI correlate with adverse brain chemistry judged by conventional microdialysis analytes, including the lactate/pyruvate ratio. Previous work on NAA has focused mainly on total tissue NAA measured via scans (in patients), or in brain tissue extracts (from animals). It is now well established that a transient decrease in brain tissue NAA is not necessarily a signal of irreversible neuronal loss. NAA loss in brain tissue appears reversible after less severe forms of brain injury.^24,31–33^ Measuring and interpreting brain tissue NAA by in vivo MRS is gaining ground as a useful non-invasive tool in patient management applicable to both severe TBI patients in parallel with invasive multimodality monitoring, as well as in less severe injury. However, scans such as these are only snapshots of the brain performed once or perhaps twice during a patient's stay in neurocritical care. Our aim in the present study has been to assess eNAA concentrations post-TBI, and determine whether or not these show a similar behavioral pattern to the total NAA measured by scanning techniques in TBI patients, or in brain tissue extracts from animal models. In addition to this, we aimed to ascertain the relationship, if any, between eNAA and established extracellular markers of brain chemistry (namely glucose, lactate, pyruvate, glutamate, glycerol, and the lactate/pyruvate ratio). The continuous nature of microdialysis means that it can be applied to monitor changes in brain energy metabolism after TBI, using time-variant information that would not be available from single time-point scans.

### Mechanism of NAA efflux immediately after TBI

It is known that the concentration of NAA in extracellular fluid is significantly lower than the tissue NAA concentration in the human brain.^34^ Previous studies using ^1^H-MRS have reported whole–brain NAA levels of approximately 12 mM.^8,35,36^ In the present study, we have detected average NAA concentrations in the range of 10–20 μM in extracellular fluid obtained from both injured and uninjured patients, suggesting a substantial NAA gradient from the intracellular to the extracellular environment. Our time-course analysis of eight patients following severe TBI by MD showed an initial elevated level of eNAA in the first day after injury, after which the concentration rapidly decreased from 24 to 75 h post-injury before stabilizing (at *∼*15 μM) from 75 h post-injury until monitoring ceased at around 120 h post-injury. Notably, the initial steep decline in eNAA concentration did not occur in patients where the MD catheter was inserted later and monitoring did not begin until 50 h+ post-injury; in the latter patients, the decline over time was considerably shallower. These results suggest that the initial high eNAA concentration in the early time-points post injury was mainly a result of the injury rather than as an artifact of catheter insertion.

In animal models subjected to controlled TBI followed by induced hypotension-hypoxia, Al Samsam and colleagues report an eNAA concentration of approximately 10 μM before injury using a microdialysis flow rate of 2 μL min^−1^.^28^ This eNAA concentration subsequently increased to approximately 80 μM at 100 min after injury before steadily declining to ∼30 μM at the end of monitoring (240 min post-injury).^28^ Based on these results, Al Samsam and colleagues suggest that at the time when monitoring ceased, the eNAA concentration was in the process of returning to the initial concentration measured prior to injury.^28^ Here, we have measured eNAA in the parenchyma of eight non-TBI patients who have had a microdialysis catheter inserted into radiographically normal brain during resection surgery of benign brain tumors, enabling a comparison to be made between the extracellular environment of TBI and non-TBI brain tissue. An average eNAA concentration of 26 μM was detected in the samples obtained from the non-TBI subjects (24 h pooled microdialysates), which is higher than the average of 17 μM obtained in TBI patients. The results obtained here suggest that the steady-state eNAA concentration reached in the days after severe TBI injury is lower than the baseline eNAA level would have been before injury, which does not agree with the theory of Al-Samsam and colleagues.^27,28^ This result is based on the assumption that the non-TBI control patients used in this study provided a true representation of healthy brain tissue.

Two mechanisms have been suggested for the increased efflux of NAA from neurons into the extracellular environment in the first few days after TBI. Firstly, direct mechanical injury caused by insertion of the MD catheter could result in a concentrated area of cellular damage. This would result in disruption of the plasma membrane of neurons in the immediate area surrounding the probe, releasing intracellular NAA into the tissue and causing a spike in eNAA levels as previously shown in animal models.^28,37^ The return to baseline levels after this kind of damage occurred ≤2 h after insertion.^28,37^ The time-scale of re-equilibration after insertion of a MD probe into human brain is not known but is likely to occur within 3–4 h after insertion. As the monitoring of eNAA was performed ≥12 h after MD probe insertion, we believe it is unlikely that mechanical injury caused by MD probe insertion is the reason for the high eNAA levels measured at earlier time-points in this study. A more likely explanation for the elevated eNAA seen here in the first 2 d after TBI is the release of intracellular neuronal NAA into the extracellular environment. This efflux of intracellular NAA could be the result of disruption of neuronal plasma membranes via physical injury, or the intracellular acidosis and cellular swelling commonly associated with ischemia, either of which would result in the uncontrolled release of NAA.^28,38,39^ Alternatively, it has been suggested that neurons could export NAA across the plasma membrane *via* active transport.^28^

### Reduced eNAA in later time-points after TBI

A decrease in the stabilized concentration of eNAA in the 50 h after TBI, as observed here, has previously been reported in animal models. Di Pietro and colleagues reported a decrease in eNAA in the 6–12 h after severe TBI in rats, followed by stable eNAA levels (∼4–5 μmol g^−1^), which were 50% to 65% lower than the average eNAA concentration in control (non-TBI) animals.^6^ This decrease in the final concentration of eNAA >75 h after injury is similar to the difference in eNAA levels we found in the present study between the TBI patients at >96 h post-injury and the non-TBI patients. Due to the lack of mortality in the small cohort of time-course patients studied here, we were unable to draw conclusions as to a possible difference in either the temporal profile or the actual values of eNAA between surviving and non-surviving patients. We also obtained no evidence to suggest that eNAA increased at later time-points in patients with poor clinical outcome, as reported by Belli and colleagues.^23^ It was noted that the concentration of eNAA was highest in the first sample taken after injury from the patient with the worst clinical outcome. This result suggests the possibility that higher eNAA concentrations immediately after injury (before the “baseline” concentration is reached) could be associated with poorer clinical outcome.

Several studies on whole–brain NAA levels measured by ^1^H-MRS also have reported decreased NAA levels in TBI patients >50 h after injury.^40^ A number of causal mechanisms have been suggested; these include widespread neuronal loss resulting in a concomitant reduction in the overall concentration of NAA present, an increase in the uptake of eNAA by glial cells due to increasing energy demands, and a decrease in the rate of NAA synthesis as a result of mitochondrial or metabolic dysfunction.^28,32,40^ In this study, we have attempted to establish the most probable of these mechanisms by studying eNAA levels in response to the addition of fuel (glucose) to the immediate extracellular environment, as well as by establishing the temporal profiles of other biomarkers in relation to eNAA in patients with TBI.

A link between ATP synthesis and the ability to synthesize NAA was reported by Bates and colleagues,^41^ and a concomitant reduction in brain tissue NAA and ATP was found in animal models of TBI and after mild TBI in humans.^6,32,42^ As much evidence suggests that NAA is synthesized in the mitochondria, the NAA depletion is thought to be a marker of metabolic impairment and the depletion of energy stores in the brain.^3,39,43^ Moffet and colleagues^39^ propose that the decrease in NAA observed after brain injury is partly the result of a reduction in synthesis due to a shortage of acetyl-CoA brought about by metabolic crisis, as well as increased degradation to provide acetate groups for lipid synthesis in myelin repair, as described by Madhavaroe and colleagues.^14^ Under normal conditions, surplus acetyl-CoA not required for ATP production via TCA cycle can be exported from mitochondria to the cytoplasm if first converted to NAA by the enzyme Asp-NAT. This cytoplasmic store of acetyl-CoA can then be shuttled to oligodendrocytes, hydrolyzed by aspartoacylase, and used for lipid synthesis and energy conversion.^44^ Under metabolic stress, a shortage of acetyl-CoA could result in reduced NAA synthesis and increased hydrolysis to provide acetate for myelin repair.^3,39^ Among other functions ascribed to NAA is the idea that it is involved in osmoregulation.^16^ Thus, release of intracellular NAA into the extracellular space may be a response to hypo-osmotic stress, although it is thought that NAA is only a minor contributor to the pool of osmotically-active molecular species in brain.^16^

The synthesis of NAA is directly correlated to neuronal metabolic state and the production of ATP. This has been confirmed by Bates and colleagues, who showed that preventing ATP production via the inhibition of enzymes of the oxidative phosphorylation pathway also led to a decrease in NAA.^41^ Previous studies have shown that artificially increasing the concentration of glucose in the extracellular fluid leads to an improvement in neuronal energy status (measured as ATP/adenosine diphosphate) after brain injury.^6^ Here we have measured eNAA levels in response to an increased glucose concentration provided in the form of glucose-enriched perfusion fluid (4 mM glucose) via the MD catheter. We show that the average eNAA in TBI patients who received the glucose-enriched perfusion fluid was not significantly different from that of the TBI control patients (who received unsupplemented perfusion fluid). A possible explanation is that the concentration of glucose used, and the localized nature of MD-based perfusion, were insufficient to provide a definitive change in the metabolic state of the surrounding neurons. This explanation appears consistent with the ISCUS results for these patients during the unsupplemented and glucose-supplemented periods, which showed an increase in microdialysate glucose concentration as expected but little change in the levels of the other ISCUS analytes.^30^ Higher concentrations of glucose, or administration over a larger area, may improve results for this type of investigation. Another possible explanation could be that the turnover of NAA is slow in brain tissue, as previously suggested by labeling studies in animals.^45^

### Relationship between eNAA and other MD analytes

The measurement of bioanalytes such as pyruvate, lactate, and glucose using microdialysate analyzers can provide information about the relative contributions of aerobic and anaerobic metabolism, cell membrane integrity and the energy status of the brain.^46^ Elevated levels of lactate and a raised L/P ratio is indicative of increased anaerobic metabolism, and acts as a reliable indicator of ischemia or hypoxia, or mitochondrial dysfunction.^30^ Ischemia is generally associated with poor outcomes in TBI patients, and can occur as a result of increased ICP and inadequate levels of brain oxygen or perfusion.^46^ With modern neurocritical care protocols, ischemia usually is avoided, although microvascular ischemia may occur in some cases.^47^ Glycerol, which is released by enzymatic cleavage of cell membrane triglycerides, is regarded as an indicator of tissue damage.^48^ In this study, a limited number of TBI patients (*n* = 14) were monitored for brain oxygen (PbtO_2_) during the microdialysis sampling period. No relationship existed between eNAA concentrations and average PbtO_2_ in this small dataset. All of the average PbtO_2_ values measured were ≥20 mm Hg, except in two patients whose average PbtO_2_ was in the high teens. None of the TBI patients monitored had PbtO_2_ averages of ≤10 mm Hg, indicating that none were badly hypoxic during monitoring.

Belli and colleagues have previously investigated the relationship between eNAA and lactate, pyruvate, glycerol and glutamate in 19 patients with TBI using MD.^23^ They report a non-recoverable fall in NAA from Day 4 after injury onwards, which coincides with increases in both the concentration of glycerol and the L/P ratio.^23^ Here, we established a significant correlation between eNAA and glycerol, pyruvate, and L/P ratio in TBI patients, in agreement with the relationships identified by Belli and colleagues.^23^ Overall, the relationship between eNAA and pyruvate and the L/P ratio confirms the involvement of NAA in the energy state of neurons, particularly in glycolysis and energy production within the cell, and is consistent with the idea that eNAA increase is linked to adverse brain metabolism. As glycerol is regarded as a marker of neuronal membrane disintegrity, the observed correlation between eNAA and glycerol suggests that the initial eNAA peak in the first 50 h after injury may result from the release of intracellular NAA into the interstitial space. The subsequent reduction in eNAA level at >75 h after injury could therefore relate to a combination of neuronal death and a reduced energy state in the remaining cells.

### Limitations

The application of microdialysis for the sampling of bioanalytes comes with the risk that an artificial signal could be generated by localized injury sustained during catheter insertion into the tissue. In this study, the monitoring of eNAA was performed ≥12 h after catheter insertion to be certain that no such artifacts were analyzed. As previously discussed, injury caused by probe insertion could result in localized neuronal death and a spike of eNAA immediately after catheter insertion.^28,37^ In the present study, we have concluded that the initially high concentrations of eNAA detected in patients monitored at the earliest time-points were not an artifact associated with catheter insertion, but rather the result of the TBI itself. This conclusion was based on the results obtained from patients for whom the monitoring began late (>50 h post-injury), where the levels of eNAA measured were within the baseline range and did not feature any significant variation. An additional limitation of the microdialysis sampling technique used is that the measurement of eNAA at time-points within 24 h of the time of injury, which would be greatly informative for this particular study, is not possible due to the time required for hospital admission and clinical treatment of patients prior to catheter insertion. As with the earlier study by Belli and colleagues,^23^ the small number of patients limits the present study; also, the potential exists (at least in theory) for variations in the microdialysis extraction efficiency for eNAA. However, because eNAA is a small water-soluble molecule (with a MW of 175 Da), recovery seems unlikely to be an issue. For future investigation, the analysis of eNAA by microdialysis sampling in combination (within the same patients) with scans and spectroscopy techniques such as ^1^H-MRS would certainly further the current findings, and provide greater scope for determining the overall function of NAA in the brain and its clinical implications.

## Conclusions

In the present study, we have analyzed the temporal profile of eNAA in eight patients with severe traumatic brain injury. We have shown that the eNAA concentration decreases rapidly in the first few days after injury, that a correlation exists between the level of eNAA and time since injury, and that the stable eNAA concentrations following the initial decline were lower in subjects with TBI than those measured in non-TBI patients. We additionally show that eNAA concentrations showed a positive correlation with glycerol and L/P ratio, and an inverse correlation with pyruvate. We have additionally measured eNAA levels in 22 other TBI patients and six tumor resection patients. We find no significant difference between eNAA levels in patients who have received microdialysis fluid supplemented with glucose and those who have not. Our findings suggest that changes in eNAA after TBI are consistent with adverse brain energy metabolism and the release of intracellular components, perhaps as a result of neuronal death or injury. Relatively high levels of eNAA correlate with poor brain chemistry, as determined using a conventional bedside microdialysis analyzer for routine analytes, including the lactate/pyruvate ratio. Extracellular NAA levels measured in microdialysates collected continuously at the bedside provide extracellular information that is complementary to that of intermittent scans with in vivo MRS, in which diminished levels of (predominantly intracellular) total brain NAA is regarded as indicative of adverse brain chemistry. Further research is merited to ascertain whether elevated levels of eNAA are a useful microdialysate biomarker of neuronal damage and disturbed metabolism, with potential applicability to patient prognosis and to monitoring of therapies. Currently, eNAA is only measurable offline in the laboratory, which limits its potential clinical utility, but in principle, development of a specific sensor for bedside use may be feasible in future.
